# Critical point drying of brain tissue for X-ray phase-contrast imaging

**DOI:** 10.1107/S1600577526001402

**Published:** 2026-03-23

**Authors:** Safe Khan, Jonas Albers, Artem Vorobyev, Yuxin Zhang, Jakob Reichmann, Angelika Svetlove, Fabio De Marco, Ksenia Denisova, Yikai Yang, Florent Seichepine, James O. Douglas, Elizabeth Duke, Peter Cloetens, Alexandra Pacureanu, Andreas T. Schaefer, Carles Bosch

**Affiliations:** a Sensory Circuits and Neurotechnology Laboratory The Francis Crick Institute London United Kingdom; b European Molecular Biology Laboratory Hamburg Unit c/o DESY Hamburg Germany; c ESRF – The European Synchrotron, Grenoble, France; d Department of Bioengineering Imperial College London London United Kingdom; e Department of Materials Imperial College London London United Kingdom; f Department of Neuroscience, Physiology and Pharmacology University College London United Kingdom; Chinese Academy of Sciences, China

**Keywords:** phase contrast, X-ray, sample preparation, neuroscience, tissue

## Abstract

X-ray phase-contrast tomography can efficiently image brain tissue at sub­cellular resolution. However, current sample preparation methods are not optimized to exploit the full potential of X-ray contrast mechanisms. Here we propose to replace interstitial material by air to enhance X-ray phase contrast of the ultrastructural features. Critical point drying (CPD) of heavy-metal-stained mouse brain tissue produced samples with preserved ultrastructure, a nanofoam-like material that remains compatible with follow-up conventional resin embedding. Using two synchrotron-based setups, namely, a high-throughput microtomography beamline and a nanoscale holographic tomography beamline, we found that CPD samples consistently showed 2–4× stronger phase-shift signal than samples embedded in resin. CPD offers a versatile route for preparing tissue for sub­cellular and ultrastructural-resolution X-ray imaging. It retains structural detail while improving signal, and is compatible with follow-up protocols involving femtosecond laser milling or electron microscopy, paving the path for biological tissue imaging beyond the mm^3^ scale.

## Abstract 2

X-ray phase contrast tomography can efficiently image brain tissue at subcellular resolution. Critical point drying allows a gentle replacement of interstitial material by air, enhancing X-ray phase contrast of the ultrastructural features.

## 1. Introduction

X-ray phase-contrast imaging can efficiently map millimeter-scale volumes of soft tissue, a size that can contain com­plete modules of neuronal circuits in the mammalian brain, at sub­cellular resolution without the need to slice the samples, by leveraging the penetrating power, contrast mechanisms and flux of synchrotron-based coherent X-ray microscopy (Kuan *et al.*, 2020 ▸; Bosch *et al.*, 2022 ▸; Bosch *et al.*, 2025 ▸; Zhang *et al.*, 2025 ▸). X-ray phase-contrast imaging (Cloetens *et al.*, 1999 ▸; McMorrow & Als-Nielsen, 2011 ▸; Salditt *et al.*, 2017 ▸; Tao *et al.*, 2021 ▸) offers the potential to bridge scales, capturing cellular and sub­cellular architecture, across intact tissue volumes with isotropic resolution approaching sub-40 nm detail (Bosch *et al.*, 2025 ▸; Laugros *et al.*, 2025 ▸). However, sample preparation protocols of biological tissues for X-ray phase-contrast imaging (Zhang *et al.*, 2022 ▸; Zhang *et al.*, 2025 ▸; Bosch *et al.*, 2022 ▸; Khimchenko *et al.*, 2018 ▸; Töpperwien *et al.*, 2018 ▸) have largely been inherited from visible light and volume electron microscopy (vEM) workflows, including the resin-embedding protocols following heavy-metal staining. While these methods provide mechanical stability and compatibility with EM, they were not tailored to enhance X-ray contrast.

**Figure 1:**
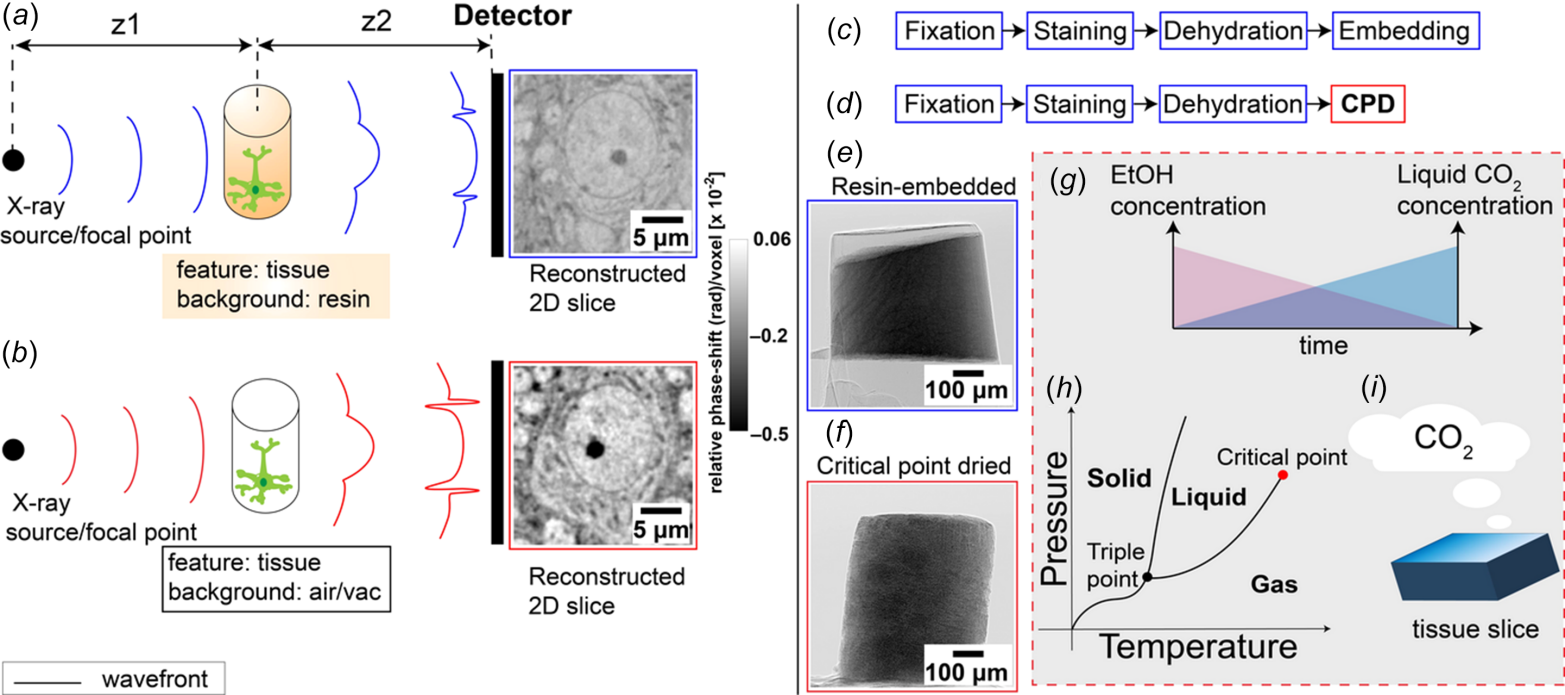
Sample preparation workflows and imaging principle for phase-contrast imaging. (*a*) Schematic of X-ray propagation through resin-embedded, heavy-metal-stained neuronal tissue. The difference in refractive index between tissue and surrounding resin causes modest distortion of the X-ray wavefront. z1 and z2 are the distances from the X-ray source or focal spot to the sample, and from the sample to the detector, respectively. (*b*) Equivalent schematic for critical point dried (CPD) tissue imaged in air or vacuum. The absence of embedding resin introduces a stronger refractive index discontinuity at the tissue–air interface, leading to enhanced wavefront distortion and increased phase contrast. (*c*, *d*) Standard preparation pipeline for Epon embedding (*c*) and CPD preparation (*d*), both following fixation, staining, and dehydration. (*e*, *f*) Representative single-projection images of femtosecond-laser-milled cylindrical pillars imaged at 17 keV in air: Epon-embedded (*e*) and CPD-prepared (*f*). (*g*)–(*i*) CPD workflow: (*g*) ethanol-to-CO_2_ exchange gradient during CPD processing, (*h*) phase diagram of CO_2_ indicating the critical point transition, and (*i*) illustration of the final CPD step where liquid CO_2_ is brought to the critical point and then slowly vented as gas while maintaining temperature, avoiding surface-tension forces associated with a liquid–gas interface and preserving ultrastructure (Williams & Clifford, 2000 ▸).

In the hard X-ray regime (5–100 keV) (Yamashita, 2003 ▸) which is typically used for imaging metal-stained biological tissues, image contrast arises from differences in refractive index between components. Upon interaction of an X-ray wavefront with matter, phase shifts distort the wavefront. Self-interference between refracted and un-refracted portions of the wavefront results in interference patterns that are measured as intensity variations after free-space propagation. Neighbouring tissue regions with different refractive indices therefore induce different phase shifts in the transmitted wavefront, generating interference patterns (or fringes) at their interfaces, which ultimately give rise to contrast in phase-sensitive imaging (McMorrow & Als-Nielsen, 2011 ▸; Salditt *et al.*, 2017 ▸; Jacobsen, 2019 ▸). In conventionally embedded tissue, contrast is generated by differences in phase shift and at­ten­uation between heavy-metal-stained structures and the sur­roun­ding carbon-rich embedding resin. Since the contrast stems from changes in electron density, one should expect to obtain enhanced contrast when replacing the interstitial material with vacuum/air instead of infiltrating it with an epoxy resin [Figs. 1 ▸(*a*) and 1(*b*)].

We therefore explored an alternative sample preparation route that could allow avoiding the embedding material altogether. Following heavy-metal staining of the mouse brain tissue samples and dehydration with a series of incubations in increasing ethanol concentrations, we used critical point drying (CPD) to remove the ethanol from the samples. This yielded a mechanically stable nanofoam-like dry scaffold in which the metal-stained ultrastructure in the tissue was surrounded by air or a vacuum, depending on the imaging requirements (Horridge & Tamm, 1969 ▸; Nordestgaard & Rostgaard, 1985 ▸; Bray, 2000 ▸; Kang *et al.*, 2018 ▸).

In CPD samples, phase contrast should be constrained by the refractive index of the foreground, as in the conventional workflow (heavy-metal-stained lipids and proteins present in plasma membranes and in protein clusters), and the refractive index of the background, which in this case should be largely minimized or negligible. This should result in more phase con­trast generated by ultrastructural features in CPD-pre­pared samples than in resin-embedded counterparts.

We first validated that the ultrastructure of CPD-treated tissue remains preserved by embedding dried samples (post-CPD) in epoxy resin and examining them with transmission electron microscopy (TEM). We then carried out X-ray phase-contrast imaging on matched sets of CPD and resin-embedded samples at two synchrotron instruments: the high-throughput imaging setup at the P14 beamline at DESY (PETRA III) and the nanoscale holographic tomography ID16A beamline at ESRF.

## 2. Results

For X-ray imaging of biological tissues to be useful for map­ping ultrastructural details and long-range cellular tracing (such as for connectomics applications), it is essential that the tissue ultrastructure remains well preserved through all stages of sample preparation. Any enhancement in phase contrast or imaging resolution is only valuable if the underlying biological features, such as cell boundaries (cytoplasmic membranes), synapses and organelles, retain their morphology. To address this, we assessed whether CPD compromises ultrastructural integrity in a way that could undermine its broader applicability. We embedded CPD-treated samples in resin, sectioned them with standard ultramicrotomy tools and imaged them using transmission electron microscopy (TEM).

Both methods to prepare samples [Figs. 1 ▸(*c*) and 1(*d*)] start with chemical fixation and heavy-metal staining (Zhang *et al.*, 2025 ▸) of tissue slices obtained from the olfactory bulb of the mouse brain (see *Methods and materials*, Section 4). These initial steps follow established protocols for brain tissue preparation for volume electron microscopy and more recently X-ray-based imaging for connectomics (Bosch *et al.*, 2022 ▸; Bosch *et al.*, 2025 ▸; Kuan *et al.*, 2020 ▸; Zhang *et al.*, 2025 ▸). For standard resin embedding, after dehydration through graded ethanol series, samples are embedded in Epon [Figs. 1(*c*) ▸ and 1(*e*) ▸]. In the CPD workflow, ethanol-dehydrated samples were transferred into liquid CO_2_ and processed through the critical point [Figs. 1(*d*) ▸ and 1(*f*)–(*h*) ▸]. CPD replaces ethanol with liquid CO_2_ [Fig. 1(*g*) ▸], which is brought to its critical point to enable drying with minimal surface-tension effects, thereby reducing the risk of damage to fragile ultrastructural features (Horridge & Tamm, 1969 ▸; Nordestgaard & Rostgaard, 1985 ▸; Bray, 2000 ▸; Kang *et al.*, 2018 ▸). The result is a dry tissue sample with preserved structural integrity but with the mechanical properties of a metallic nanofoam (Cojocaru *et al.*, 2022 ▸) [Figs. 1(*f*) ▸ and 1(*i*) ▸, and Fig. S1 in the supporting information]. Finally, after com­pleting the respective preparation workflows for resin-embedded and CPD-treated tissue, cylindrical pillars of varying diameters were fabricated using femtosecond laser milling (Bosch *et al.*, 2023 ▸) [Fig. S2 and Tables S1 and S2 in the supporting information].

**Figure 2:**
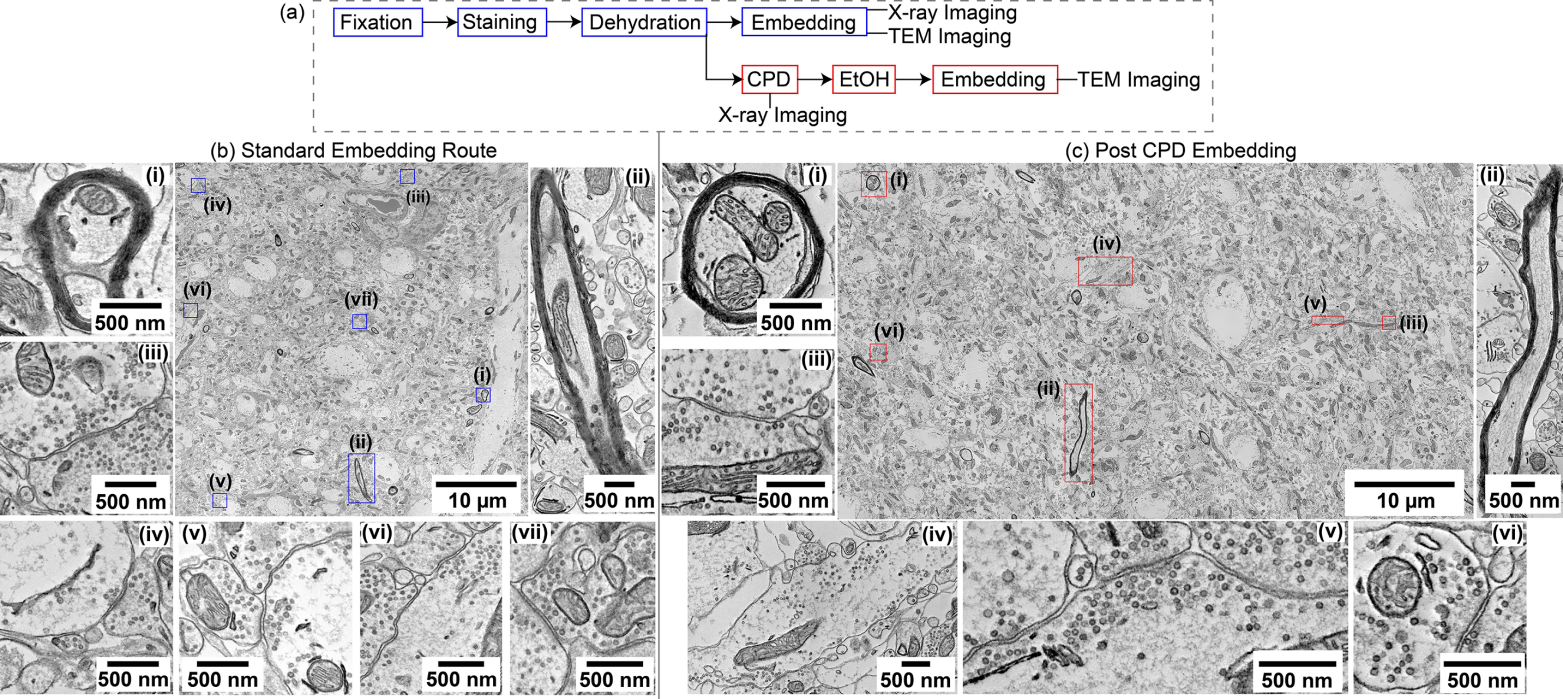
Ultrastructural preservation following critical point drying and post-CPD embedding in resin. (*a*) Divergent preparation workflows for TEM: tissue is either embedded directly in resin (top) or dried using CPD and subsequently embedded (bottom) prior to ultramicrotomy and TEM imaging. (*b*) TEM images of standard resin-embedded tissue show well preserved ultrastructure, including clear nuclear membranes, myelinated axons, and synaptic vesicle populations. (*c*) CPD-treated tissue post embedded in resin retains qualitatively comparable morphological fidelity, with intact sub­cellular features throughout. The anatomical locations of the TEM sections used in this figure are shown in Fig. S4, and additional representative TEM images from independent regions are shown in Fig. S5.

We then assessed whether CPD would compromise the preservation of ultrastructure. We stained samples of mouse brain tissue, dehydrated them and dried them with CPD as described above. At that point, we infiltrated the dried sample with 100% resin. This provided a resin-embedded specimen fully com­patible with TEM [Fig. 2(*a*) ▸]. This approach allowed us to isolate the effects of CPD on the tissue while keeping fixation, staining and dehydration protocols unaltered. Post-CPD-embedded samples displayed a preserved sub­cellular architecture [Fig. 2(*c*) ▸], largely comparable to conventionally prepared samples [Fig. 2(*b*) ▸]. Qualitatively, in both the standard and post-CPD-embedded tissue, myelinated axons, mito­chon­dria, nuclear envelopes, synaptic vesicle clusters and fine neurites remained morphologically intact. A technical re­plicate of this experiment is provided in Fig. S3 (see sup­porting information), highlighting the reproducibility of this workflow across samples. These results confirm that CPD of a dehydrated biological soft tissue largely preserves the underlying ultrastructure.

**Figure 3:**
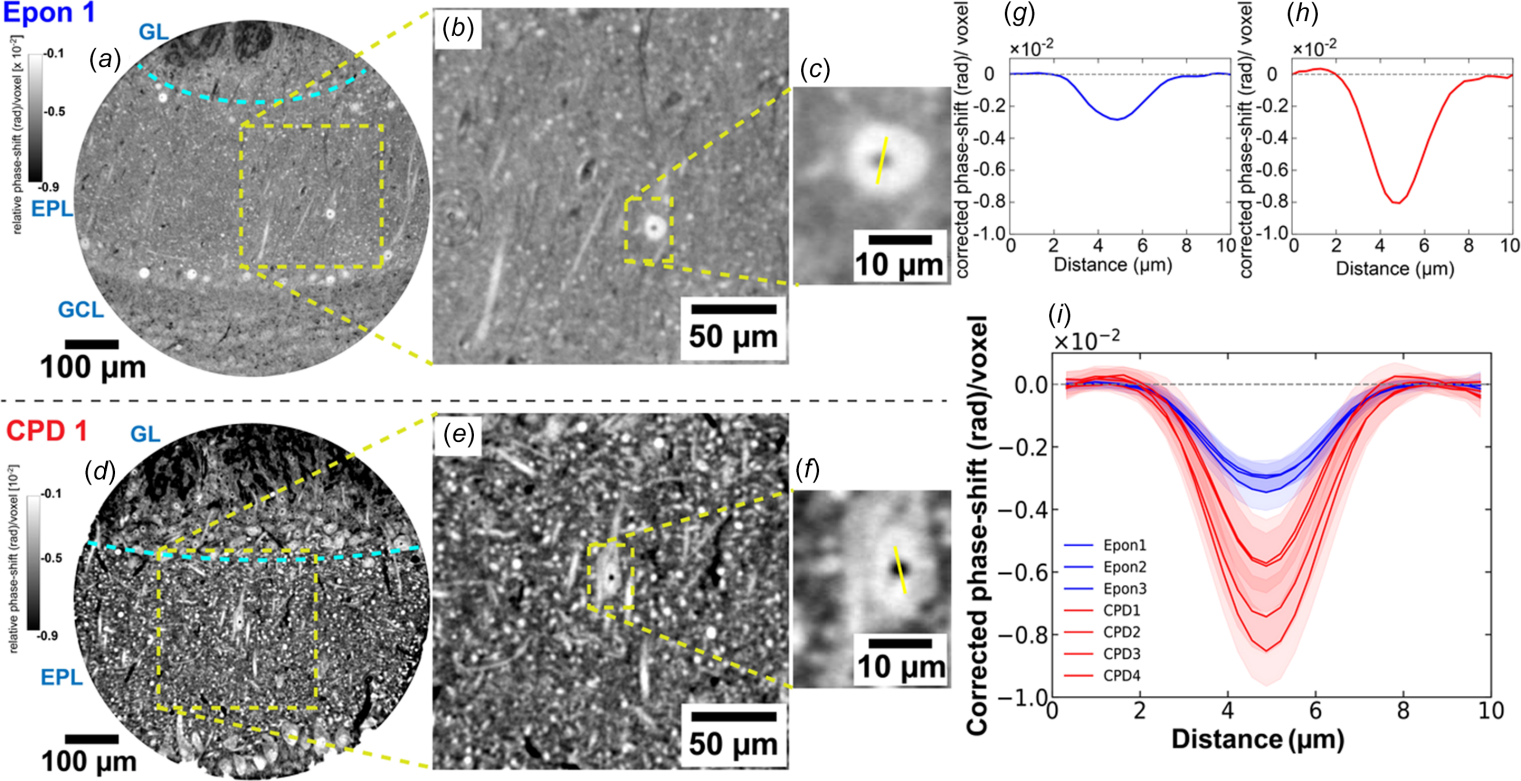
Enhanced phase contrast in CPD-treated tissue imaged *via* propagation-based XPCT at P14. (*a*)/(*d*) Reconstructed tomographic slices from embedded (Epon) and CPD-treated tissue pillars, respectively, centred on the external plexiform layer (EPL) of the olfactory bulb, and parts of the glomerular layer (GL) and granule cell layer (GLC) are also visible. (*b*)/(*e*) Magnified views within the EPL. (*c*)/(*f*) Showing nucleolus features (yellow line: profile axis), with corresponding corrected phase-shift profiles in parts (*g*) and (*h*). (*i*) Overlay of line profiles from multiple samples containing the nucleolus feature {Epon: blue, *n* = [25, 25, 25] nucleoli; CPD: red, *n* = [25, 25, 25, 25] nucleoli)}, demonstrating consistent enhancement of phase-shift [corrected phase-shift = raw-signal − baseline mean (background)] in CPD samples. Shaded areas indicate standard deviation across measured regions. Reconstructed tomographic slices of embedded and CPD datasets labelled here are shown in Figs. S6 and S7, respectively. Other individual line profiles from each dataset are shown in Fig. S8. Increased phase contrast in CPD tissue results from the higher refractive index mismatch between the tissue and the surrounding air/vacuum, compared to resin-embedded preparations.

We next evaluated how CPD preparation influences image quality in X-ray propagation-based phase-contrast tomography (XPCT) of tissues. We wanted our quality metric to be sensitive to the sample preparation method but resilient to sample and region-specific variability. Image contrast satisfied this requirement, since it allowed for image acquisition regimes compatible with acquiring sample replicates with fields of view sufficiently large to target the same histological layer in all cases. We imaged (in air at an energy of 17 keV) CPD and resin-embedded samples at the EMBL High-Throughput Tomography (HiTT) setup of the P14 beamline (PETRA III), which employs a four-distance propagation-based acquisition scheme (Albers *et al.*, 2024 ▸). In both sample types, we could easily identify the biological features, such as cell bodies, nucleoli, apical dendrites of projection neurons and glomeruli, which are commonly resolved in 325 nm effective pixel size XPCT datasets (Bosch *et al.*, 2022 ▸). CPD samples however showed improved phase contrast in all those features [Figs. 3(*a*) ▸ and 3(*d*) ▸]. To quantify this effect, we moni­tored signal in a sub­cellular feature that is widely distributed in all samples and that provides a consistently sharp contrast: the nucleolus. This sub­cellular compartment is located inside the nucleus, in the shape of a spheroid of approximately 2 µm in diameter in principal neurons (Hieber *et al.*, 2016 ▸), and is mainly composed of proteins and nucleic acids. Accordingly, like other protein clusters (such as postsynaptic densities), it accumulates heavy metals during the staining protocol. Importantly, its immediate surrounding, the nucleoplasm, has a much lower density of lipids and proteins, resulting in a sharply defined, compact and frequent feature that contains the limits of the dynamic range of signal in the dataset. Therefore, nucleoli, visible as small dark cores within the pale nucleus of mitral or tufted cells, the principle neurons of the mouse olfactory bulb, provided an excellent substrate for a comparative quantitative analysis of X-ray phase con­trast signal.

We extracted the image intensities of the voxels following a line that crossed the nucleolus across multiple nucleoli in each analysed tomogram [Figs. 3(*b*)–3(*c*) ▸ and 3(*e*)–3(*f*) ▸] and corrected the signal obtained by subtracting the baseline measured in the adjacent nucleoplasm to robustly monitor the phase-shift across the nucleolus in CPD and Epon-embedded samples [Figs. 3(*g*) and 3(*h*)]. CPD samples consistently exhibited deeper intensity troughs [Figs. 3(*g*), 3(*h*) and 3(*i*)]. The en­hanced signal we observed in CPD samples reflects a stronger phase contrast triggered by ultrastructural features. Furthermore, a similar enhancement of X-ray scattering was also observed in unstained samples processed *via* CPD (*i.e.* aldehyde fixation is followed by dehydration without any staining step) when compared to unstained samples embedded in paraffin (Fig. S9). Together, these results suggest that CPD preparation causes the enhancement in X-ray phase shift that leads to increased contrast in the images, independently of the staining method being used.

To assess whether the contrast improvements seen in CPD-prepared tissue extend into the nanoscale imaging regime, we next imaged CPD-prepared samples with X-ray holographic nanotomography (XNH) at the ID16A beamline (ESRF). This imaging modality offers higher spatial resolution and increased coherence compared to propagation-based phase contrast. We configured scans using an isotropic voxel size of 100 nm, which in turn allowed obtaining single tiles of fields of view extending 300 µm in diameter. The ability to propagate wavefronts over extended distances in this configuration increases sensitivity to subtle phase gradients which enables the resolution of finer structural features. Imaging was per­formed under vacuum at 33.6 keV.

**Figure 4:**
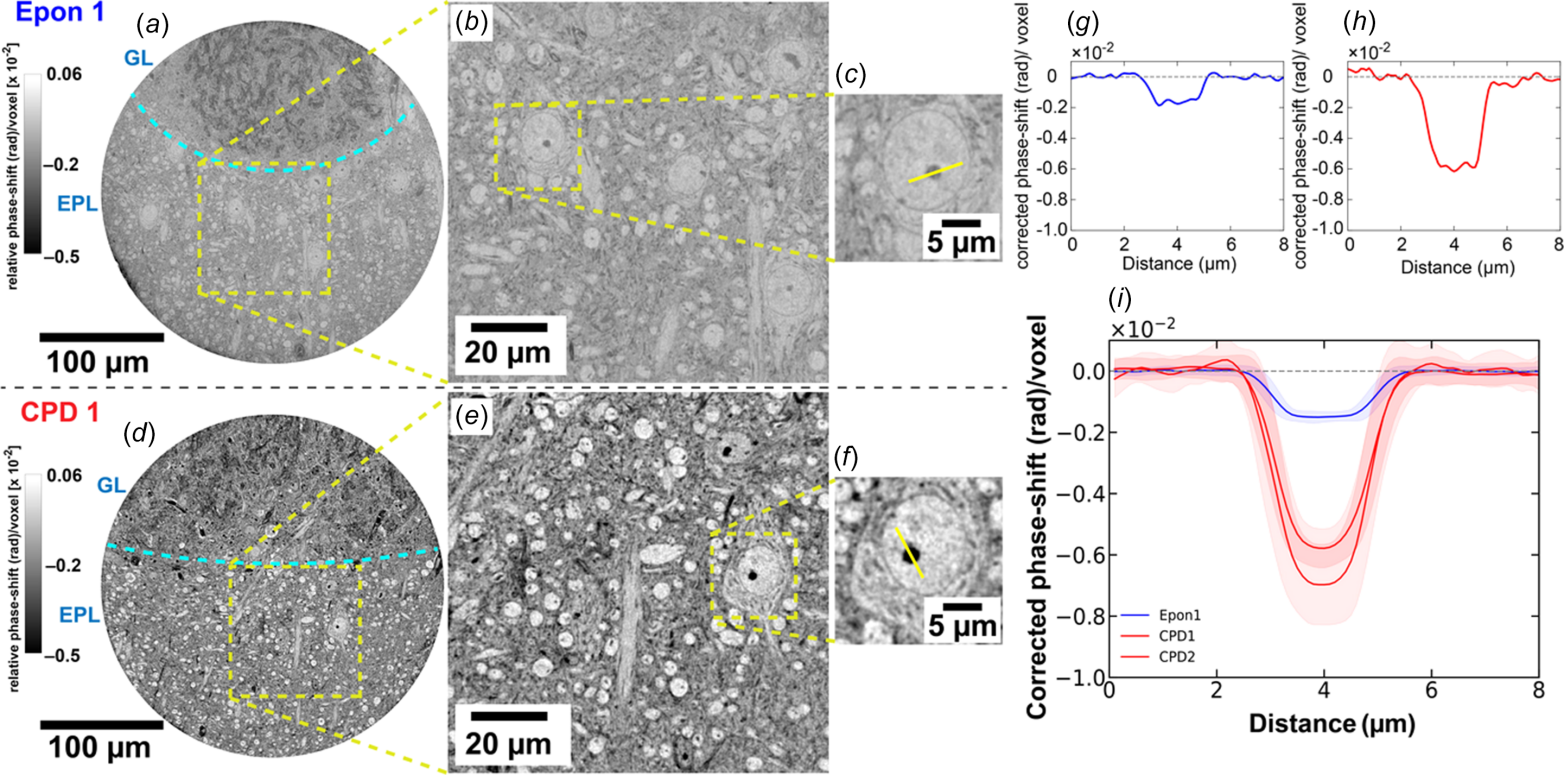
Enhanced phase contrast in CPD neuronal tissue imaged via XNH at ID16A. (*a*)/(*d*) Reconstructed slices from embedded (Epon) and CPD tissue, respectively, imaged in the external plexiform layer (EPL) and glomerular layer (GL) of the mouse olfactory bulb. (*b*)/(*e*) Magnified views within the EPL. (*c*)/(*f*) Showing nucleolus features (yellow line: profile axis), with corresponding corrected phase-shift profiles in parts (*g*) and (*h*). (*i*) Averaged line profiles from multiple nucleoli in Epon (blue, *n* = 100 nucleoli) and CPD (red, *n* = [100, 50] nucleoli) samples, illustrating enhanced phase modulation [corrected phase-shift  = raw-signal − baseline mean (background)] and gradient sharpness in dried tissue. Shaded bands represent standard deviation. Individual line profiles from each dataset is shown in Fig. S10.

Both resin-embedded and CPD samples were obtained from a targeted and well characterized anatomical region of the olfactory bulb, containing the external plexiform layer (EPL), as well as the adjacent glomerular layer (GL) [Figs. 4(*a*) ▸ and 4(*d*) ▸]. As in the P14 datasets, CPD samples dis­play stronger phase contrast across both broad tissue interfaces and fine sub­cellular structures.

We quantified phase contrast in these datasets, as before, by characterizing the signal amplitude across nucleoli [Figs. 4(*b*)–4(*c*) ▸ and 4(*e*)–4(*f*) ▸]. Line profiles drawn across these features [Figs. 4(*g*) ▸ and 4(*h*) ▸] revealed an enhanced phase shift in CPD samples [Fig. 4(*i*) ▸].

**Figure 5:**
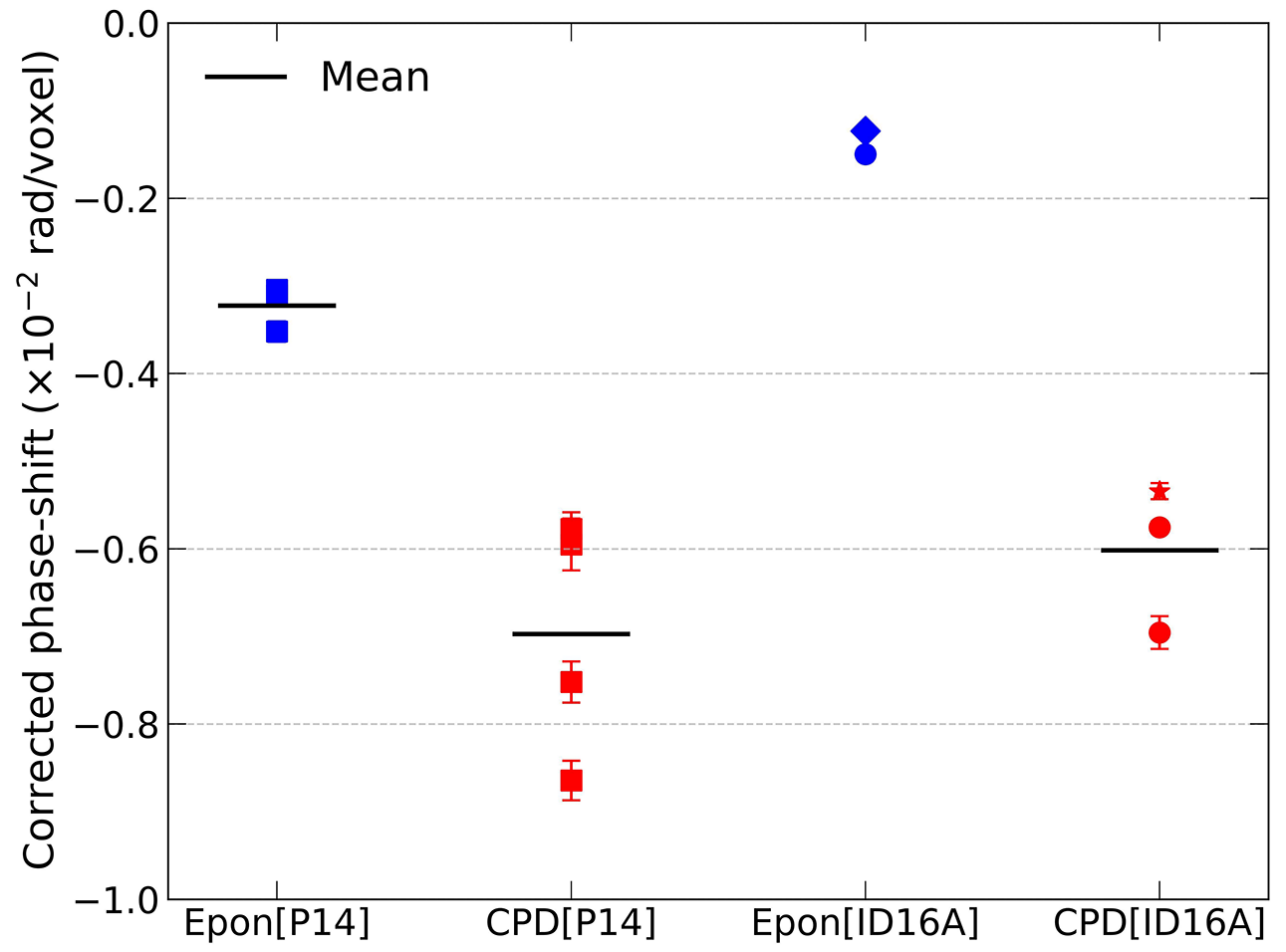
Summary of phase-shift enhancement across all datasets and imaging modalities. Amplitude of the corrected phase-shift (rad/voxel), extracted from averaged line profiles across nucleolar features in CPD-prepared (red) and Epon-embedded (blue) neuronal tissue. Data points include measurements acquired at P14 (squares) and ID16A (circles). Special cases (measured at ID16A) are marked: star indicates a different brain region (thalamus) and smaller pillar diameter (150 µm), as shown in Fig. S11; diamond indicates a dataset collected under different imaging conditions, as detailed in Fig. S12. Despite differences in voxel size and imaging geometry, CPD consistently yields stronger phase-shift signals, confirming the generality of phase contrast enhancement introduced by CPD. Horizontal black bars indicate the mean value for each group.

Overall, our findings show that CPD preparation of brain tissue samples introduces stronger X-ray scattering compared to standard resin-embedding protocols. CPD samples, devoid of any embedding material, exhibit a higher refractive index difference between foreground – lipids and proteins – and background – cytosol – increasing the contrast of tissue ultrastructure. We further compared the background-corrected phase shift amplitudes across all samples and beamlines explored (Fig. 5 ▸). CPD samples exhibit a 2–4× increase in phase contrast compared to their resin-embedded counterparts, an effect that can also be detected in unstained samples (Fig. S9).

## 3. Discussion

We present a method to prepare brain tissue samples optimized for X-ray phase-contrast imaging. By converting tissue samples into a nanofoam-like structure, without any embedding material, the difference in scattering in sub­cellular and ultrastructural imaging is constrained between air/vacuum (little/no scattering) and that provided by the membranes (which might be stained with heavy metals of choice).

We observe a 2–4× increase in background-corrected phase shift in high-signal biological features (nucleoli). This effect is consistent not only across samples but also across X-ray phase-contrast imaging regimes and setups, as well as in unstained samples, suggesting that it might apply as well to other X-ray phase-contrast regimes such as X-ray ptychography.

This enhancement in phase contrast has meaningful implications for imaging efficiency. X-ray imaging dwell time depends quadratically on the inverse of the background-corrected signal amplitude (Du *et al.*, 2021 ▸). Therefore, a 2–4× enhancement in signal should translate into a 4–16× speed-up in imaging. Hard X-ray imaging has demonstrated its capacity to resolve features at sub-10 nm detail (Aidukas *et al.*, 2024 ▸) and ultrastructure in brain tissues below 40 nm detail (Bosch *et al.*, 2025 ▸; Laugros *et al.*, 2025 ▸) non-destructively, making it an emerging technology suitable for scaling up connectomics to the whole mouse brain scale (Jefferis *et al.*, 2023 ▸; Helmstaedter, 2026 ▸). In this context, CPD preparation of biological tissues can provide a boost in imaging speed, helping to bridge the current technological gap towards mm^3^–cm^3^-scale X-ray connectomics.

Sample ultrastructure remained largely preserved through­out CPD and follow-up resin embedding, as revealed by TEM, with neurites, synapses and vesicles displaying comparable patterns to the control specimens. Furthermore, femtosecond laser milling (Bosch *et al.*, 2023 ▸) provided a unique tool to manipulate the resulting metallic nanofoam samples and to generate targeted pillars of controlled diameter (150–1000 µm). Together, this highlights a new tech­nological framework for preparing tissue samples for ultrastructural imaging that carries multiple advantages. First, since this protocol eliminates the need of any resin infiltration step, it could bring a robust alternative to prepare samples of mm^3^–cm^3^ in volume (Song *et al.*, 2023 ▸; Lu *et al.*, 2023 ▸). In practice, the scalability of CPD for biological specimens is primarily governed by solvent-exchange kinetics and the physical dimensions of the CPD chamber, rather than by the critical point drying principle itself. Previous studies have demonstrated CPD of whole-organ specimens while preserving mesoscopic tissue architecture at the 10–100 µm scale (Krenkel *et al.*, 2014 ▸; Hagen *et al.*, 2015 ▸), indicating that the approach is compatible with mm^3^–cm^3^ volumes. Second, this method uncouples the choice of resin from the X-ray phase-contrast imaging step. Therefore, this enables CPD-prepared mm-scale tissues to be optimally imaged with X-rays before trimming them into arrays of 200 µm-wide samples with a femtosecond laser, which can then be individually embedded in the optimal resin for their targeted imaging endstation. Neighbouring tissue regions could therefore meet the standards to be imaged with follow-up Focused Ion Beam SEM (by, for example, embedding that one pillar with durcupan) and with serial block-face SEM (by, for example, embedding that other pillar with Epon). And third, CPD sample preparation combined with whole mouse brain staining methods, femtosecond laser milling and high-throughput imaging approaches [such as those offered by high-throughput sub­cellular-resolution synchrotron X-ray phase-contrast beamlines (Albers *et al.*, 2024 ▸; Bonnin *et al.*, 2024 ▸; Mittone *et al.*, 2022 ▸; Tuieng *et al.*, 2025 ▸)], offers a versatile and high-throughput workflow that can help scale up the bandwidth of tissue life science nano-imaging experiments.

While this study focused on improving background-corrected signal by reducing the background, further improvements may come from optimizing staining protocols, particularly through the use of lighter elements with favourable X-ray scattering properties. These optimizations will likely provide further improvements in contrast and therefore in imaging speed, paving the way for tissue-scale X-ray connectomics. Moreover, the optimized preparation of unstained biological tissues for X-ray imaging can broaden the reach of tissue nanoimaging to scientific cases where heavy-metal staining might pose a limitation. This advantage might therefore appeal to studies on unconventional animal species, exploring diverse developmental stages, or in locations where the logistics of heavy-metal manipulation are difficult to implement – such as in fieldwork or in clinical setups aimed at exploring the volume ultrastructure of solid biopsies.

Finally, we report a versatile approach to create nanofoams with customized properties, using aldehyde-fixed biological tissues as a source material. These nanofoams can be tuned to match requirements by staining the fixed tissues with the metals of choice prior to dehydration. This approach is therefore compatible with optimizing tissue staining to maximize X-ray phase contrast, but also to match requirements for other applications in which nanofoams might be of use.

Altogether, our findings show that removing interstitial material from biological tissues through CPD enhances X-ray phase-contrast signal by 2–4×. This boost can, in principle, translate into a larger improvement in imaging efficiency, providing a means to scale-up X-ray phase-contrast imaging workflows of biological tissues without compromising ultrastructural integrity.

## 4. Methods and materials

### 4.1. Sample preparation

All animal protocols were approved by the Ethics Committee of the board of The Francis Crick Institute and the United Kingdom Home Office under the Animals (Scientific Procedures) Act 1986.

A summary of all samples can be found in Tables S1 and S2 in the supporting information.

*Dissection and fixation.* Mice were sacrificed and 300–500 µm thick coronal brain sections containing the olfactory bulb (OB) region were cut using a Leica VT1200S vibratome. Slicing was per­formed in an ice-cold dissection solution containing 65 m*M* NaH_2_PO_4_·H_2_O (phosphate buffer), 4.6% (146 m*M*) sucrose, 0.6 m*M* CaCl_2_ and 0.02% sodium azide (adjusted to an osmolarity of 300 ± 20 mOsm/L), bubbled with 95% O_2_/5% CO_2_ to adjust the pH, to keep the tissue moistened and cold throughout dissection and slicing. Immediately following sectioning, tissue was transferred into ice-cold fixative, consisting of a mixture of 1.25% glutaraldehyde and 2.5% paraformaldehyde in 150 m*M* sodium cacodylate buffer (pH 7.40, 300 ± 20 mOsm/L). Samples were incubated in fixative overnight at 4°C. The next day, tissues were washed three times for 10 min each in wash buffer (150 m*M* sodium cacodylate, pH 7.40, 300 ± 20 mOsm/L) at 4°C. All steps were carried out in ice-cold osmolarity-verified buffers to ensure tissue preservation.

*Staining.* Samples were processed using a standard heavy-metal protocol (rOTO) (Zhang *et al.*, 2025 ▸). Tissue was incubated in 2% osmium tetroxide (OsO_4_) in 0.15 *M* sodium cacodylate buffer (NCB) for 1.5 h at 20°C, followed immediately by 2.5% potassium ferrocyanide in 0.15 *M* NCB pH 7.40 for an additional 1.5 h at 20°C, without an intermediate water wash. Samples were then treated with 1% thiocarbohydrazide (aqueous) for 45 min at 30°C, followed by a second osmication step using 2% OsO_4_ (aqueous) for 3 h at 20°C. Afterwards, samples were incubated in 1% uranyl acetate (aqueous) overnight at 4°C and later warmed to 50°C for 2 h. Lead aspartate staining (aqueous, pH 5.0) was per­formed for 2 h at 50°C. Water washes were carried out between each staining step unless stated otherwise.

*Resin embedding.* Following staining, samples were dehydrated through a graded ethanol series (75, 90, 100 and 100%), transitioned into propylene oxide. After this step, one set of samples underwent infiltration with increasing concentrations of hard Epon resin [Epon812 (TAAB T023), DDSA (TAAB D026), MNA (TAAB M011) and BDMA (TAAB B008), mixed as described by Bosch *et al.* (2025 ▸) and Glauert & Lewis (2014 ▸)] diluted in propylene oxide (25, 50, 75, 100 and 100%). Resin-infiltrated samples were polymerized for 72 h at 60–70°C. One of these samples (C417Fb) was embedded in a similar resin that used a different epoxy, EMbed812 (EMS 14900).

*Critical point drying.* Critical point drying (CPD) followed directly after ethanol dehydration. CPD was employed to preserve tissue morphology by avoiding the damaging effects of surface tension that arise during conventional air drying. In hydrated biological specimens, evaporation at the liquid–air interface can introduce substantial tangential forces, leading to deformation, collapse or delamination of micro- and nanoscale structures (Horridge & Tamm, 1969 ▸; Nordestgaard & Rostgaard, 1985 ▸; Bray, 2000 ▸; Kang *et al.*, 2018 ▸). CPD circumvents this by transitioning from the liquid to the gaseous phase without crossing a distinct phase boundary. Because the critical point of water (374°C, 229 bar) is incompatible with biological preservation, water was first replaced with a series of intermediate exchange solvents, ethanol in this case, which is miscible with both water and liquid CO_2_. Ethanol was then gradually exchanged with liquid CO_2_. Unlike ethanol or acetone, which require extreme conditions for CPD (critical points above 230°C), CO_2_ reaches its critical point at 35°C and 70 bar, making it suitable for biological applications. Once in the CPD chamber (Leica EM CPD300), the liquid CO_2_ was brought to its critical point and vented slowly by reducing the pressure while maintaining the critical temperature, thus drying the sample minimizing phase transition forces. The parameters used for CPD on the Leica EM CPD300 were as follows: CO_2_ In (speed = slow, fillers = full, delay = 120 s), Exchange (speed = 5, cycles = 14) and Gas Out (heat = medium, speed = slow / 100%), resulting in a total process duration of approximately 100 min.

*Resin embedding after CPD.* Dried samples were incubated with 100% ethanol for 4 h at 4°C (changing ethanol at every hour mark) and polymerized following the standard resin-embedding procedure.

*Femtosecond laser milling.* Both sets of resin embedded and dried tissue samples were then milled to cylindrical shapes with a femtosecond laser (Optec Femtosecond laser WS Starter equipped with a Coherent Monaco laser with dual wavelength, 515 and 1030 nm; all pillars in this study are milled with a wavelength of 515 nm). The diameters of the final cylinders were decided so samples would fit in the field of view of the tomography experiments at synchrotron beamlines. Pillars with diameters of ∼500 and ∼300 µm were milled for measurements at P14 and ID16A, respectively (Fig. 1 ▸ and Fig. S2).

### 4.2. Transmission electron microscopy

To confirm ultrastructure preservation, transmission electron microscopy (TEM) experiments were per­formed on thin sections of ∼80 nm thickness (obtained *via* Ultramicrotome sectioning) in both resin-embedded and post-CPD-embedded samples. A JEOL 1400FLASH electron microscope was used for this purpose. Beam current was set to 120 kV and imaging was per­formed at 12k magnification (giving a pixel size of 1.4 nm), and the JEOL LLP software suite was used for image acquisition and stitching to cover a wide region of interest (Fig. 2 ▸, and Figs. S3 and S5).

### 4.3. SEM imaging

Low-magnification block-face images (Fig. S4) were acquired using a Thermo Fisher Scientific QUANTA FEG SEM operated in low-vacuum mode (30 Pa) to minimize charging of resin-embedded samples. Imaging was per­formed at an accelerating voltage of 2.5 kV with a dwell time of 10 µs using the vCD backscattered electron detector, providing stable contrast for large-area overview imaging of tissue architecture.

### 4.4. Plasma focused ion beam (FIB) milling

Plasma FIB milling was carried out on a Thermo Fisher Scientific Helios Hydra using 30 kV Xe^+^ ions which has a current range between 1 pA and 2.5 µA. SEM imaging was carried out using using the Ion Conversion Electron Detector (ICE) in secondary electron detection mode [Fig. S1(*a*) 10 kV, 100 pA; Fig. S1(*c*) 2 kV, 100 pA; Fig. S1(*e*) 2 kV, 100 pA], the Through Lens Detector (TLD) in Field Free secondary electron mode [Fig. S1(*b*) 2 kV, 100 pA], and the Through Lens Detector (TLD) in Immersion mode with backscatter electron mode [Fig. S1(*d*) 2 kV, 100 pA]. Cross-sectional milling at 30 kV was per­formed using a range of beam currents (60, 200, 500 and 1000 nA) to identify the fastest approach for achieving a relatively undamaged surface finish. It was found that 500 nA was sufficient to quickly mill the cross sections shown in Figs. S1(*b*) and S1(*c*). 12 kV Xe^+^ ions were used to deposit a 10 µm × 10 µm × 1 µm Pt/C protective layer over a region of interest to protect the surface from milling during the shape of a pillar structure.

### 4.5. X-ray phase-contrast tomography at P14

X-ray phase-contrast tomography was per­formed at the High-Throughput Tomography (HiTT) endstation of the EMBL beamline P14 at PETRA III (DESY), optimized for in-line phase-contrast imaging of mm-sized biological samples. Brain tissue pillars were mounted vertically on custom 3D-printed holders and scanned at ambient pressure and room temperature. Each tomographic scan used four sample-to-detector distances to enable multi-distance phase retrieval (Cloetens *et al.*, 1999 ▸). All experiments at P14 were per­formed at an X-ray energy of 17 keV. A high-resolution detection system (Optique Peter) equipped with an LSO:Tb on YbSO scintillator (thickness 8–10 µm) and a 20× magnifying microscope objective was coupled with a PCOedge 4.2 sCMOS camera (2048 × 2048 pixels, 6.5 µm pixel size, maximum 100 Hz frame rate). This resulted in an effective pixel size of 0.325 µm, yielding a field of view of 666 µm × 666 µm, which was sufficient to cover the full pillar diameter (500 µm) in a single scan. The acquisition covered 181° of rotation with 1800 projection angles. The raw intensity projections at each distance were flat-field corrected, aligned and subsequently processed using a non-iterative contrast transfer function (CTF)-based phase-retrieval algorithm (where the refractive index decrement δ and absorption index β were set to that of osmium, and a δ/β value of 10 was approximated for an X-ray energy of 17 keV) (Cloetens *et al.*, 1999 ▸). After retrieval, projections were subjected to tomographic reconstruction using the gridrec (regridding reconstruction) algorithm to obtain volumetric datasets. The HiTT pipeline also includes on-the-fly data reduction and speed-optimized multi-node phase retrieval and tomographic reconstruction (Nikolova *et al.*, 2024 ▸), which allows for real-time feedback of reconstructed tomogram images to image acquisition parameters, enabling an efficient handling of multiple samples (Albers *et al.*, 2024 ▸). The X-ray energy at P14 is tunable; however, to ensure a consistent comparison between resin-embedded and CPD samples, all measurements were per­formed at the same energy. We selected 17 keV as the standard operating energy at the HiTT endstation, offering a practical balance between phase-contrast sensitivity and transmission for millimetre-scale tissue.

### 4.6. X-ray holographic nanotomography at ID16A

X-ray holographic nanotomography (XNH) was per­formed at the ID16A beamline of the European Synchrotron (ESRF, Grenoble, France). The beamline’s endstation is located at 185 m from the source to improve spatial coherence and full-field nanoscale imaging is achieved thanks to nanofocusing with multilayer-coated Kirkpatrick–Baez mirrors (Cesar da Silva *et al.*, 2017 ▸). We imaged at 33.6 keV with a focal spot size of around 15 nm. Sample pillars were mounted on a high-precision rotation stage within a vacuum chamber maintained at 10^−7^ mbar. The detector was placed outside the vacuum chamber at about 1.289 m from the focal spot. For each com­plete acquisition, we acquired four tomographic scans over 180° (2000 projections each), at different propagation distances. For each rotation angle, the four corresponding holograms were flat-field corrected, brought to the same magnification and aligned in order to combine them for obtaining a phase map. Phase retrieval was then carried out using an iterative multi-distance approach, starting from an approximation regularized with a δ/β ratio of 27 (corresponding to Os at 33.6 keV) (Yu *et al.*, 2018 ▸), and only the phase term was updated per iteration while keeping the amplitude constant. Retrieved phase maps were used for tomographic reconstruction using a filtered back-projection algorithm to generate isotropic 3D volumes at 100 nm voxel size (for all datasets). The resulting datasets allowed for cellular feature discrimination across entire pillar volumes (Kuan *et al.*, 2020 ▸; Livingstone *et al.*, 2025 ▸; Zhang *et al.*, 2025 ▸; Laugros *et al.*, 2025 ▸). Due to the different X-ray energies and phase-contrast regimes, hence different phase-retrieval algorithms, at P14 and ID16A, the absolute magnitude of the recovered phase is not expected to be identical across the two instruments. For cross-instrument comparison, the ID16A phase output (cm^−1^) was converted to an effective rad/voxel scale by accounting for wavelength scaling and voxel length (see repository notebook; Khan *et al.*, 2025 ▸). To assess potential radiation-induced effects during XNH, selected resin-embedded and CPD samples were imaged repeatedly under identical acquisition conditions and with varying exposure times, resulting in increasing cumulative dose. Representative reconstructed slices from repeated scans, shown in Figs. S13 and S14, reveal no qualitative changes in tissue morphology or contrast within the explored dose range.

### 4.7. Data analysis

We used *Fiji* (Schindelin *et al.*, 2012 ▸) to obtain grey values from voxels at regions of interest. Retrieved data were then structured, analysed and plotted using custom scripts in Python. All reconstructed datasets were then stored in OME-ZARR format into a webknossos environment (scalable minds; Boergens *et al.*, 2017 ▸) for data-sharing purposes (see *Data availability* section).

## Supplementary Material

Supplementary Figures S1 to S14. Supplementary Tables S1 and S2. DOI: 10.1107/S1600577526001402/wuz5001sup1.pdf

## Data Availability

All 3D-reconstructed tomograms reported in this study and tables containing source data for all quantitative analyses are accessible through the associated code repository. Analysis code is available from https://github.com/safekhan/CPDtissuesXray.
