# Supplementary material for: Critical point drying of brain tissue for X-ray phase-contrast imaging
Source: J Synchrotron Radiat. 2026 Mar 23;33(Pt 3):759–68. doi: 10.1107/S1600577526001402 (PMC13148618; doi:10.1107/S1600577526001402)
Supplement: Supplementary file 1 [file s-33-00759-sup1.pdf]

JOURNAL OF SYNCHROTRON RADIATION

Volume 33 (2026)

Supporting information for article:

Critical point drying of brain tissue for X-ray phase-contrast imaging

Safe Khan, Jonas Albers, Artem Vorobyev, Yuxin Zhang, Jakob Reichmann, Angelika Svetlove, Fabio De Marco, Ksenia Denisova, Yikai Yang, Florent Seichepine, James O. Douglas, Elizabeth Duke, Peter Cloetens, Alexandra Pacureanu, Andreas T. Schaefer and Carles Bosch

Supporting Information

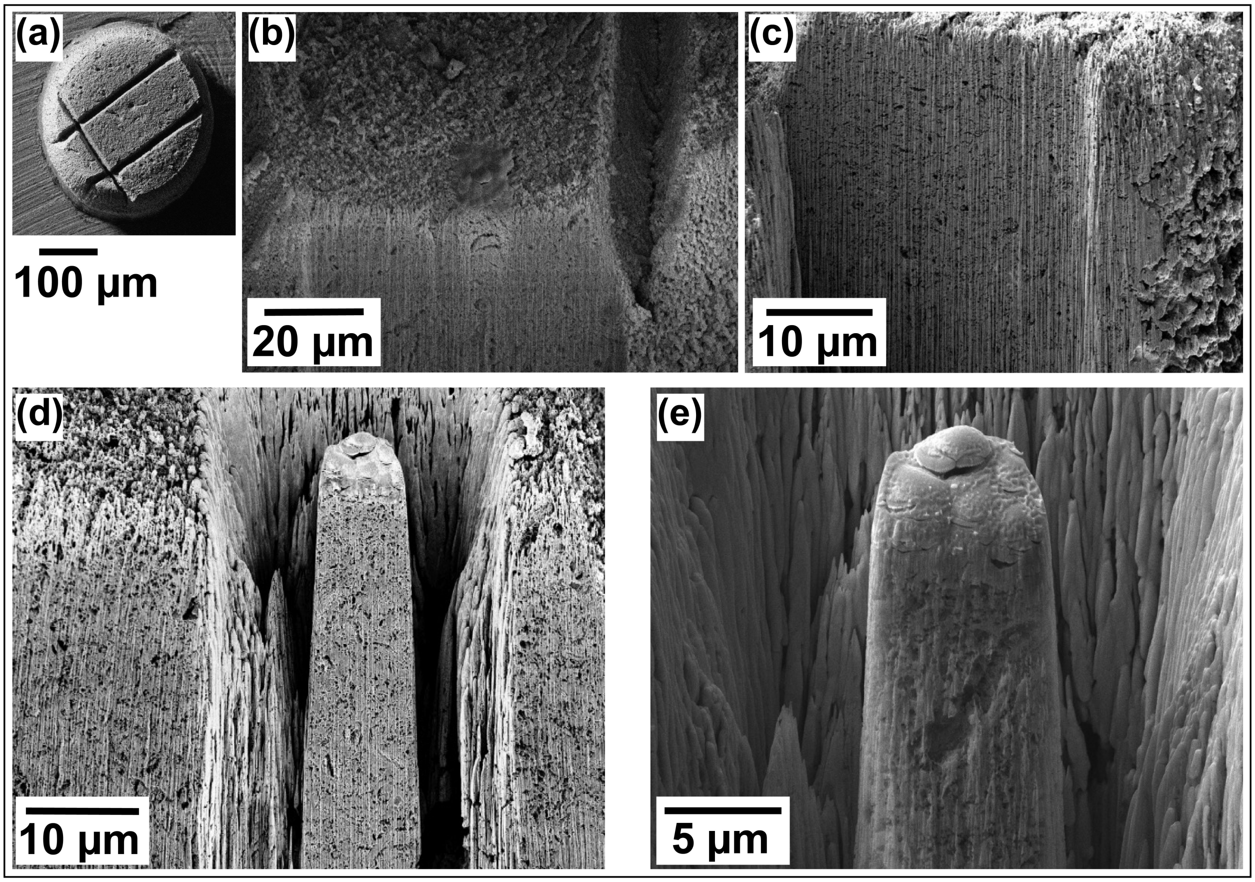

Figure S1. Plasma-Focused Ion Beam (p-FIB) milling of a CPD-prepared, metal-stained brain tissue sample reveals porous, nanofoam-like structure. (a) Top view SEM image of a ≈ 300 µm diameter CPD pillar prepared by femtosecond laser milling from a CPD-treated sample. **(b-c)** Higher magnification SEM images of the same pillar after targeted edge milling using xenon plasma-focused ion beam (p-FIB). The striated and porous appearance across the milled cross-sections reflects the air-filled nanostructure characteristic of a metallic nanofoam, consistent with the absence of embedding material. **(d-e)** On the same CPD pillar described in (a-c) we sculpted a smaller ≈ 10 µm diameter pillar using p-FIB milling.

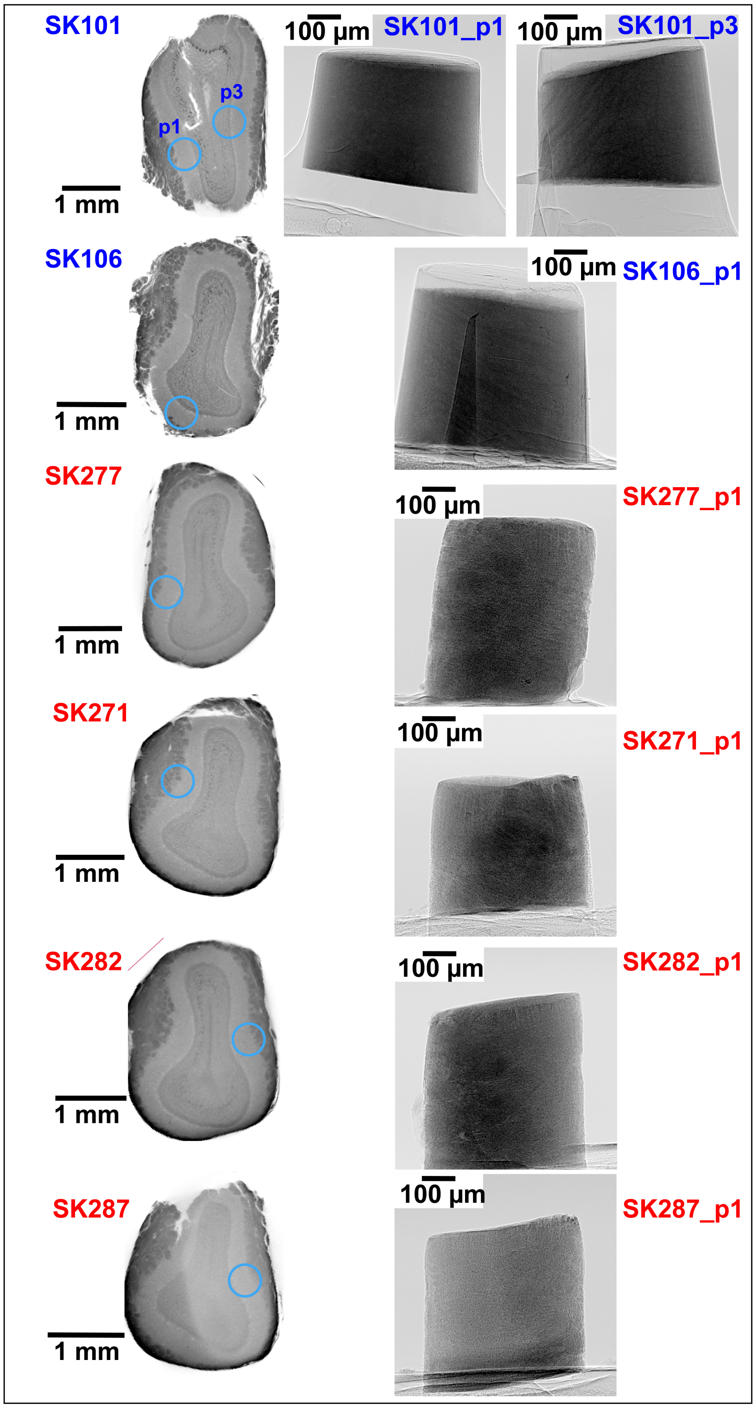

Figure S2. Overview of pillar locations for all samples imaged at P14. (a) Lab-based X-ray micro-CT scans (left) show coronal views of tissue slices from which pillars were extracted using femtosecond laser milling. Blue circles indicate the targeted regions where pillars were milled. Each slice corresponds to either resin-embedded (SK101, SK106; blue labels) or CPD-prepared (SK277, SK271, SK282, SK287; red labels) tissue. Corresponding single-projection images (right) acquired at P14 highlight pillar morphology and mounting geometry for each sample.

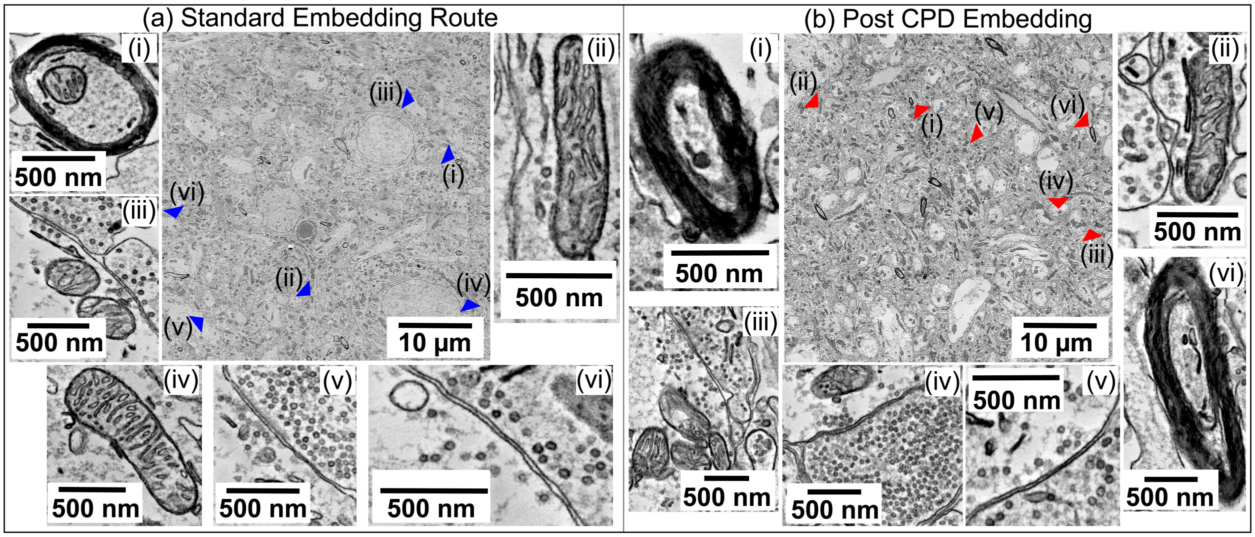

Figure S3. Consistent ultrastructural preservation in post-CPD embedded tissue across independent samples. Transmission electron microscopy (TEM) images from resin-embedded **(a)** and post CPD embedded tissue **(b)**, prepared from separately processed brain sections. Across both conditions, qualitatively, key cellular features such as mitochondria, vesicle pools, nuclear membranes, and synaptic boutons remain structurally intact.

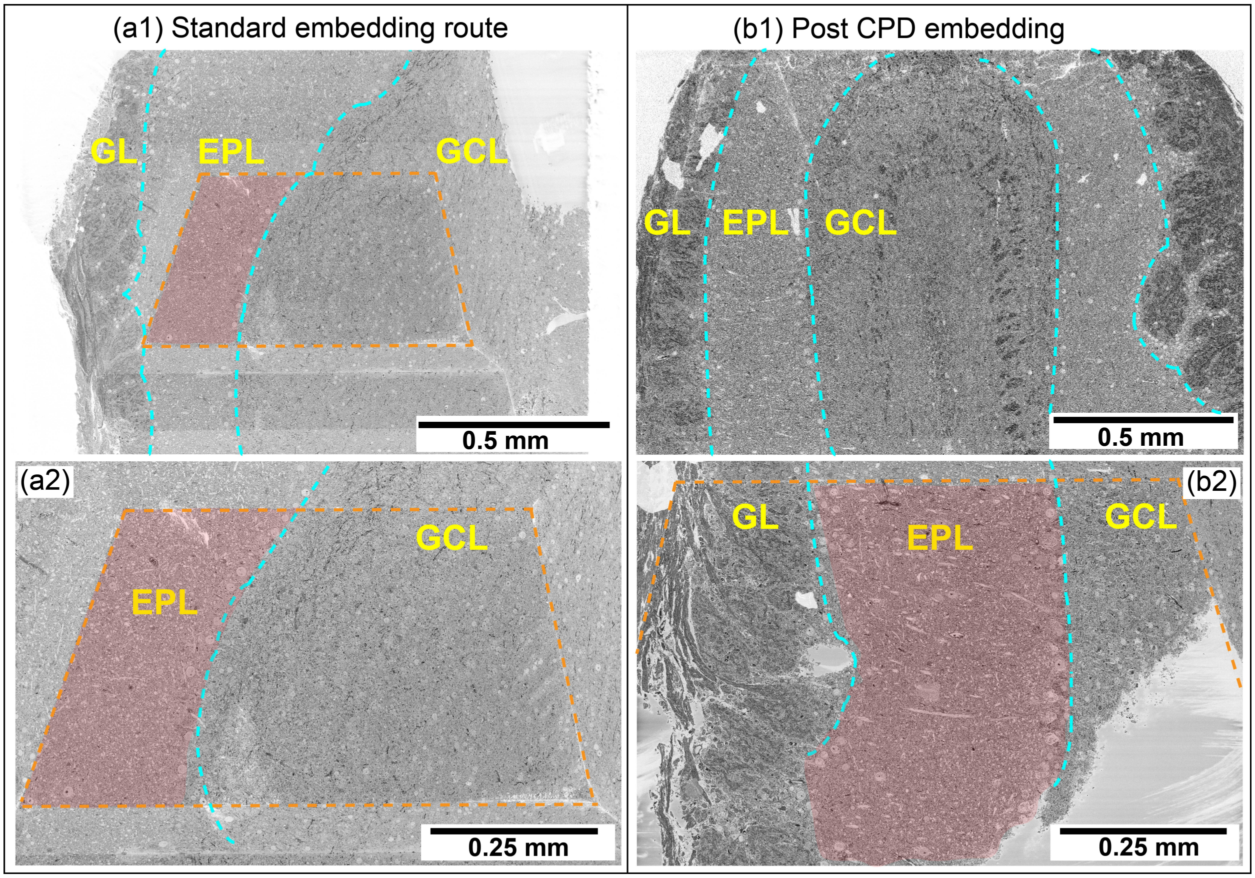

Figure S4. Location of TEM sections used for ultrastructural comparison in Fig. 2. Low-magnification blockface images of resin-embedded olfactory bulb samples prepared via the standard embedding route **(a1-a2)** and post-critical-point-drying (CPD) embedding **(b1-b2)**. Dashed cyan outlines indicate the boundaries between major histological layers (glomerular layer, GL; external plexiform layer, EPL; granule cell layer, GCL). Shaded regions highlight the portion of the external plexiform layer from which the TEM image snippets shown in Fig. 2b and Fig. 2c were extracted.

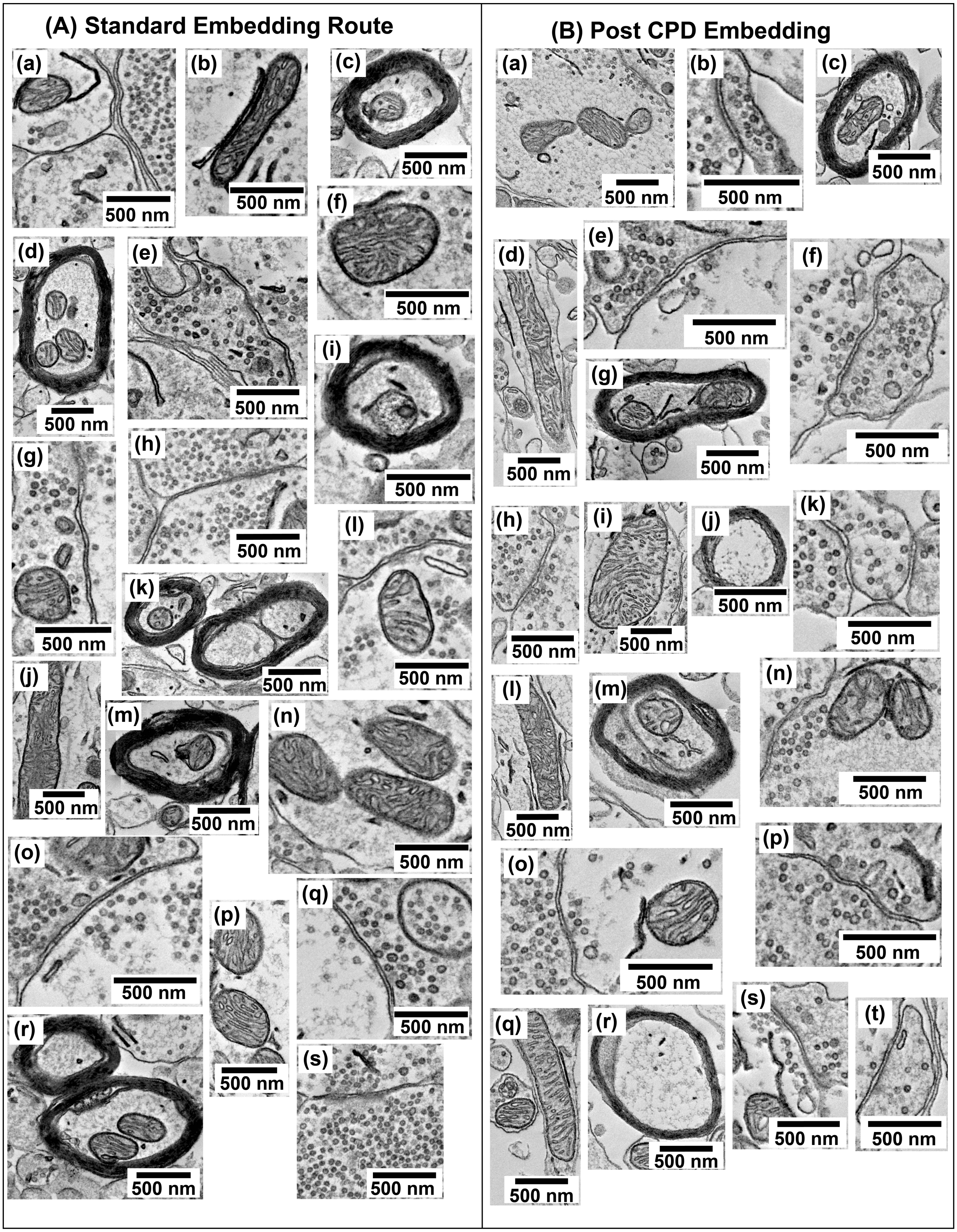

Figure S5. Preservation of ultrastructure following post-critical-point-drying embedding. Transmission electron microscopy (TEM) images comparing **(A)** standard resin embedding and **(B)** post-critical-point-drying (CPD) embedding of mouse olfactory bulb tissue, acquired from comparable regions of the external plexiform layer (EPL). Representative ultrastructural features, including membranes, mitochondria, synaptic vesicles and myelinated axons, are shown.

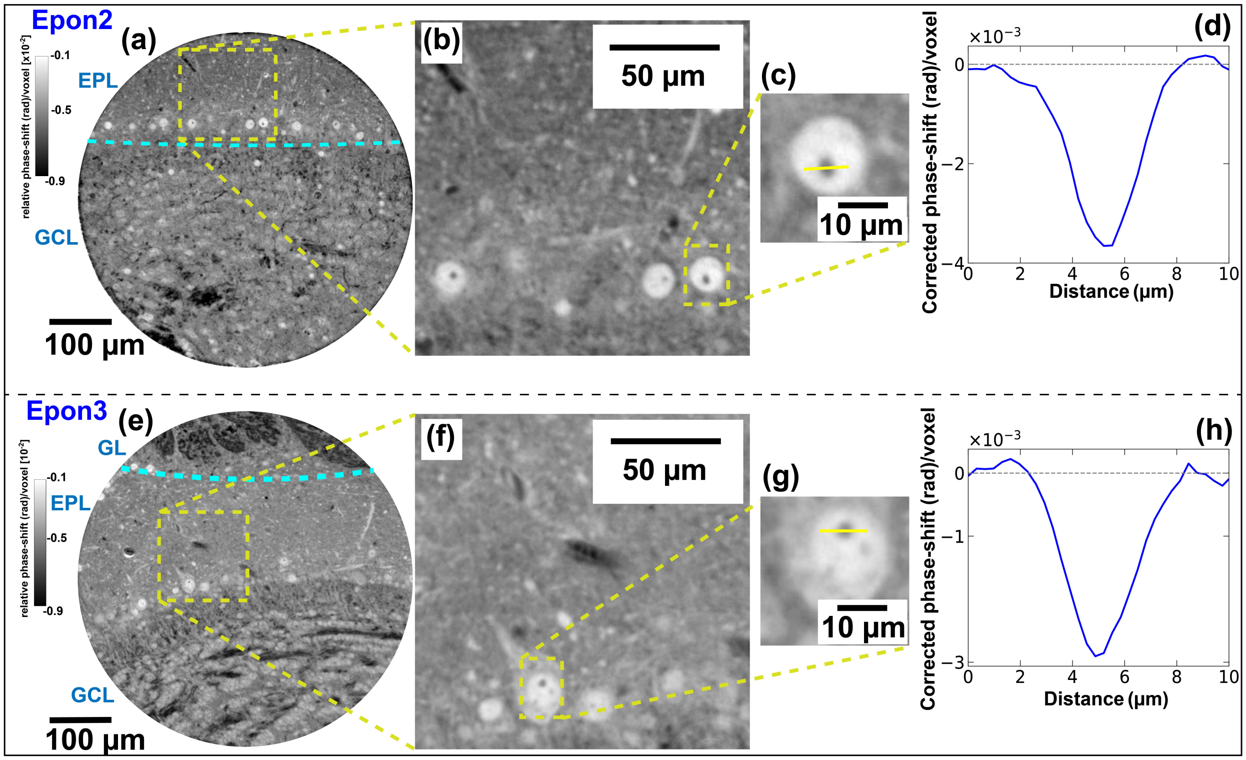

Figure S6. Examples of reconstructed slices of additional Epon-embedded samples imaged at P14 using X-ray phase contrast tomography. **(a-d)** Image and representative signal trace of sample labelled as Epon2 in the Fig 3 (i). **(e-h)** Image and representative signal trace of sample labelled as Epon3 in the Fig 3 (i).

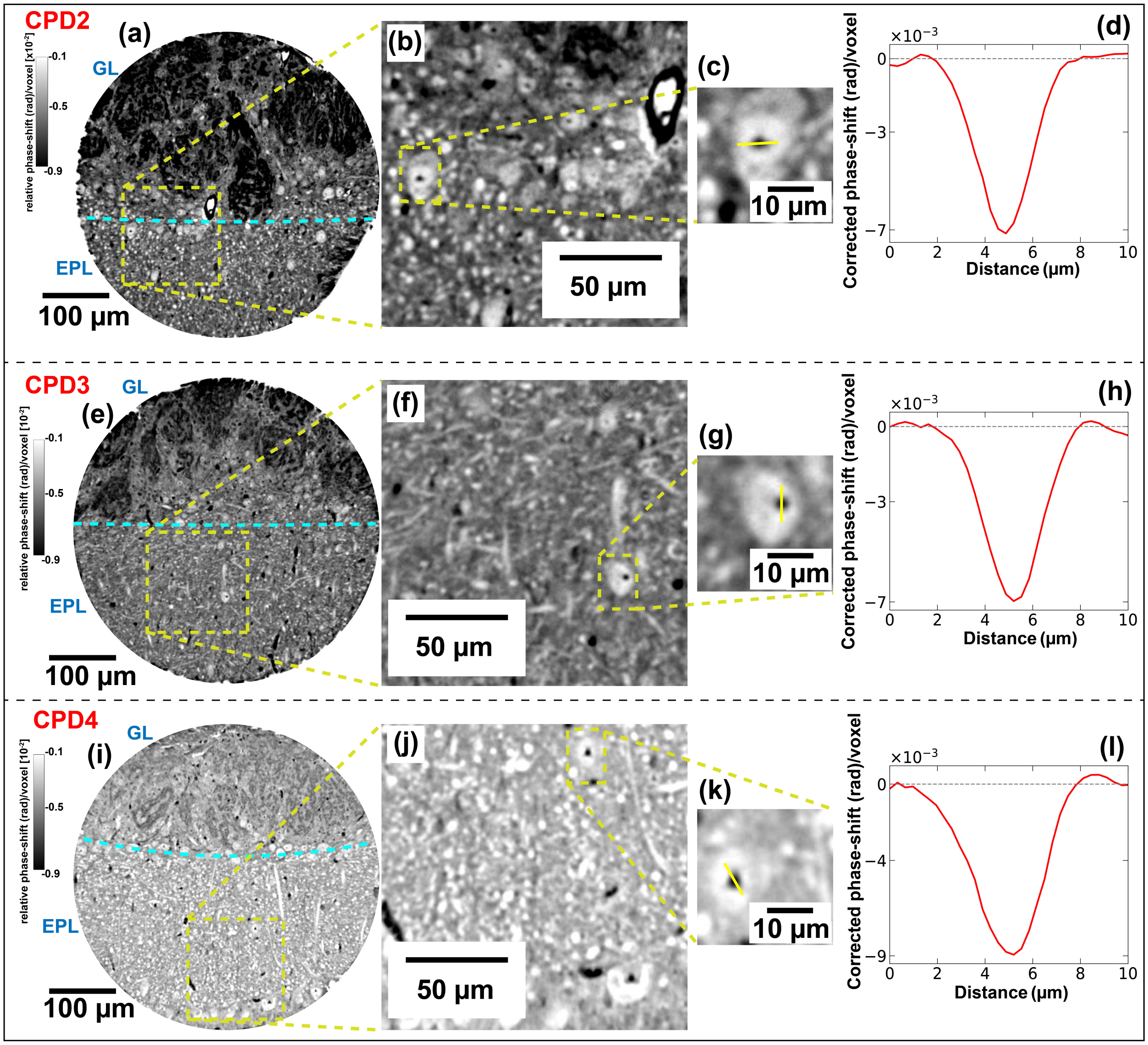

Figure S7. Examples of reconstructed slices of additional CPD samples imaged at P14 using X-ray phase contrast tomography. **(a-d)**, **(e-h)** & **(i-l)** are images and representative signal traces of samples labelled as CPD2, CPD3, CPD4 in Fig 3 (i) in the main text.

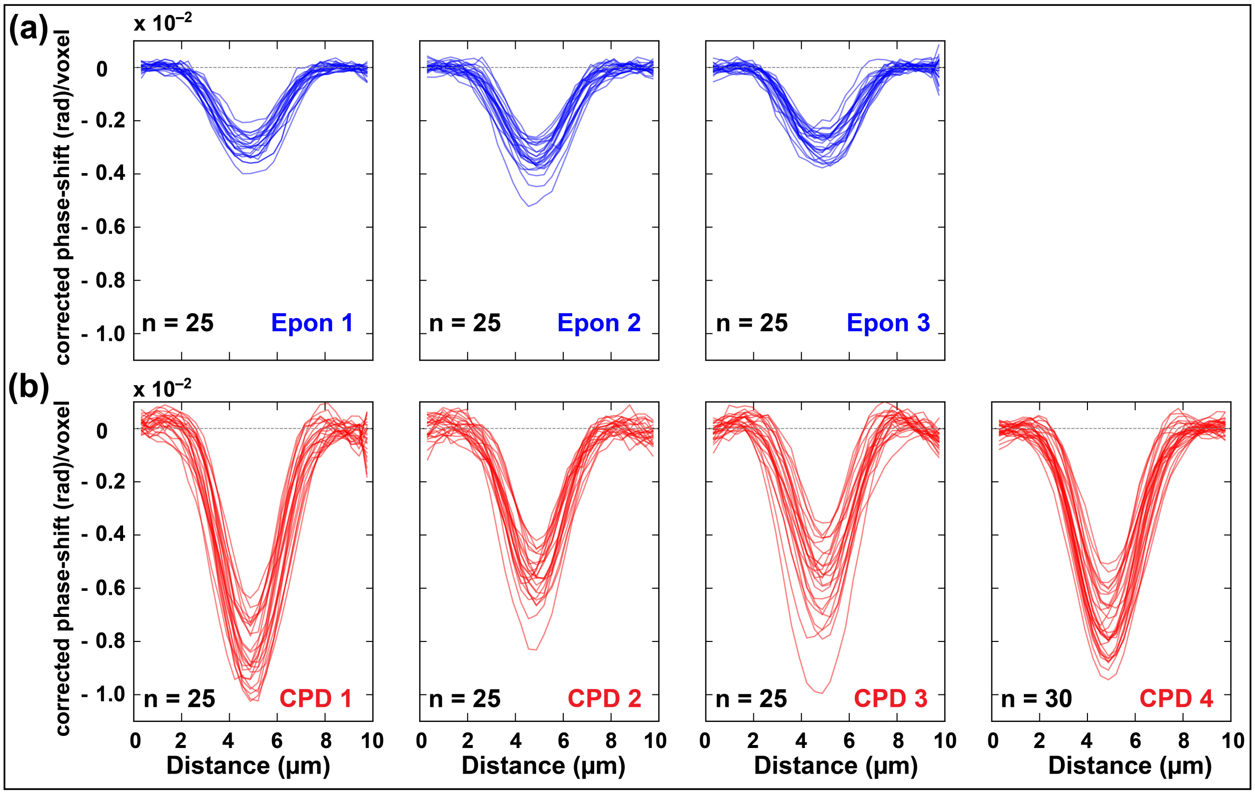

Figure S8. Background corrected line profiles for all the samples imaged at P14 using X-ray phase contrast tomography. Corrected phase-shift = raw signal − baseline mean (background). Line profiles were extracted across nucleoli (where n = number of line profiles) from multiple Epon **(a)** and CPD **(b)** datasets. The averaged line profiles from these datasets are shown in Figure 3i.

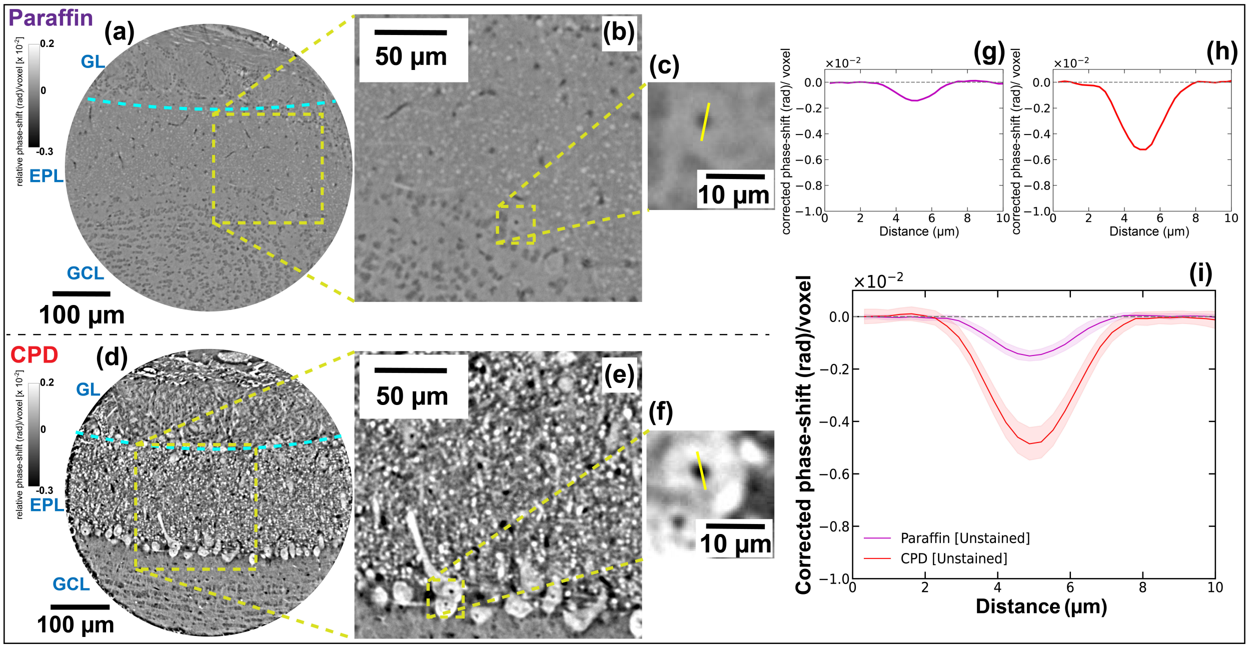

Figure S9. X-ray phase contrast signal in unstained tissue, CPD vs paraffin-embedded. Tomographic slices from mouse brain olfactory bulb tissue samples prepared without any staining and processed via either CPD or standard paraffin embedding, measured using XPCT at P14. **(a–c)** Paraffin-embedded sample. **(d–f)** CPD-prepared sample. **(g-h)**Representative line profiles in both samples. Mean ± s.d. of multiple (n=25) measurements in this dataset.

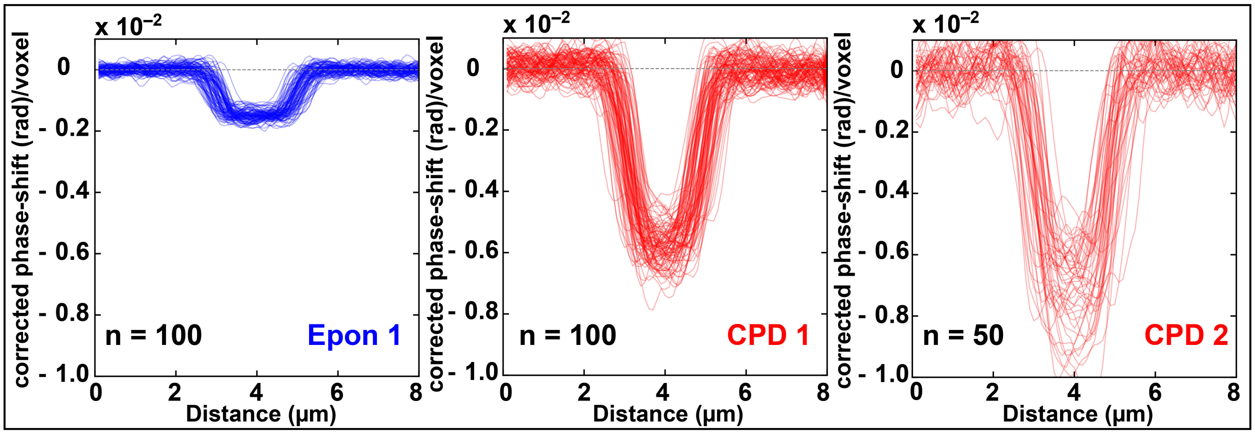

Figure S10. Background corrected line profiles for all the samples imaged at ID16A using X-ray nano-holotomography. Corrected phase-shift = raw signal − baseline mean (background). Line profiles were extracted across nucleoli (where n = number of line profiles) from Epon (blue) and CPD (red) datasets. The averaged line profiles from these datasets are shown in Figure 4i.

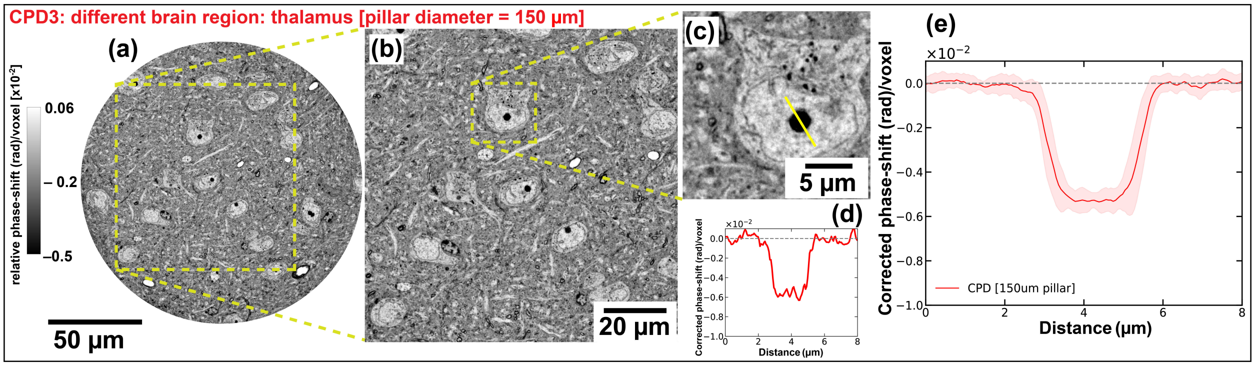

Figure S11. XNH of CPD-prepared sample from a different brain region (thalamus) and imaging configuration. (a) Reconstructed phase slice from a CPD-prepared sample imaged at ID16A (ESRF) from the thalamic region, using a 150 μm diameter pillar. The voxel size was 60 nm, and exposure time was 150 ms. **(b-c)** Zoom-in views reveal preserved ultrastructure, including cellular and subcellular compartments. **(d)** Line profile extracted across a nucleolus (yellow line in c), showing strong phase contrast at the nuclear boundary. **(a)** Mean ± s.d. of multiple (n=25) measurements in this dataset. This dataset contributes one of the CPD data points in Figure 5.

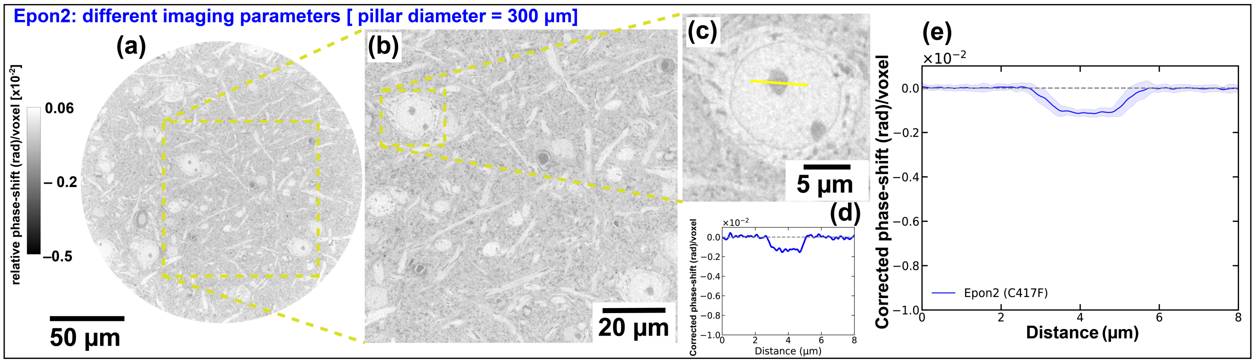

Figure S12. XNH of resin-embedded tissue (C417F) imaged with different parameters at ID16A. (a) Reconstructed phase slice of an Epon-embedded olfactory bulb sample imaged at ID16A. This scan was acquired with a 60 nm pixel size. **(b-c)** Zoom-in views of the same region highlight subcellular structures including cell somata and nucleoli. **(d)** A representative line profile across a nucleolus (yellow line in c) shows low phase contrast, consistent with reduced signal due to refractive index matching between tissue and resin. **(e)** Mean ± s.d. of multiple (n = 25) measurements in this dataset. This dataset contributes an additional data point in Figure 5.

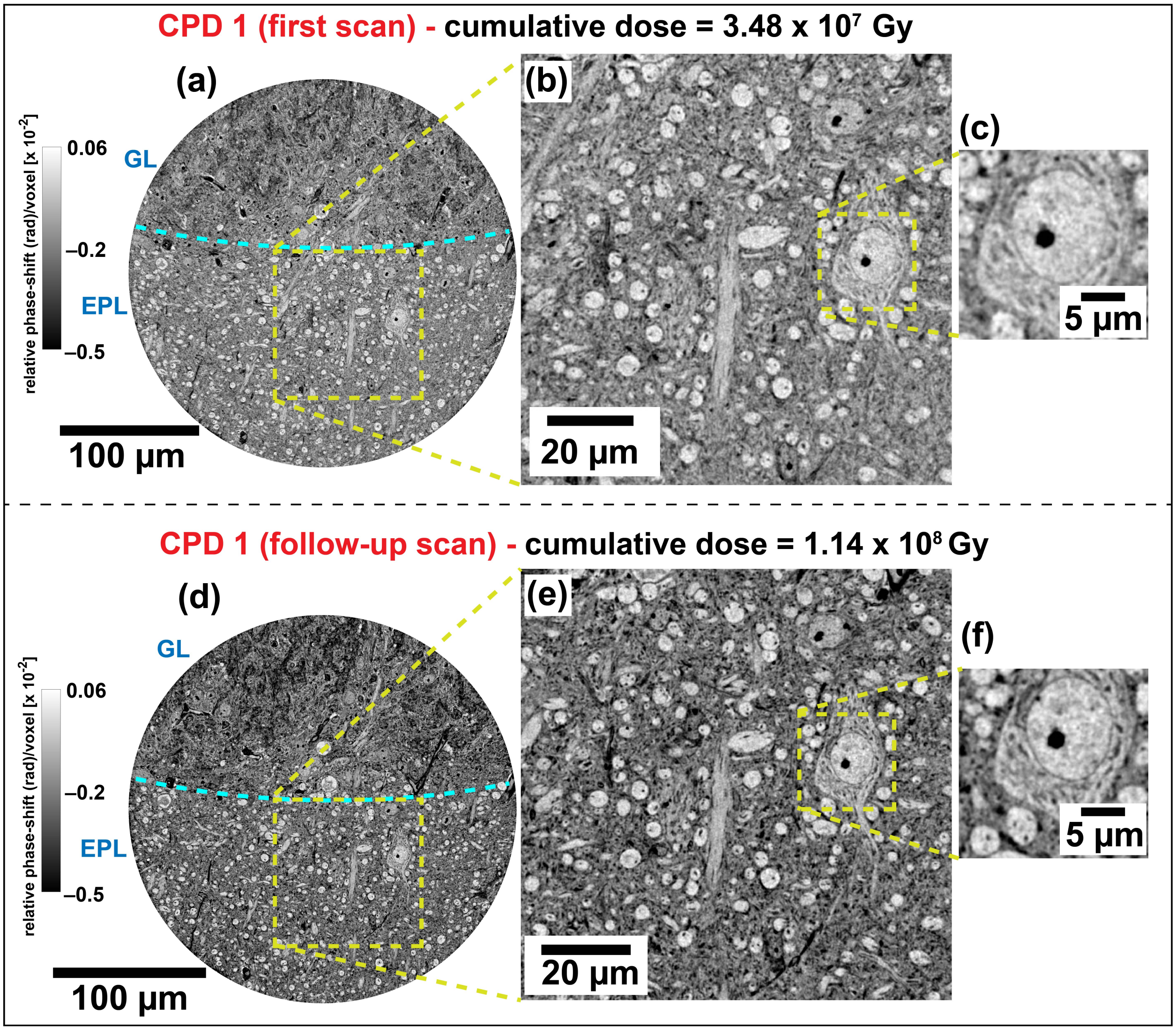

Figure S13. Radiation dose tolerance under repeated holotomographic imaging of critical-point-dried brain tissue. Representative reconstructed phase-contrast slices from a critical-point-dried (CPD) brain tissue sample (SK185_p1) acquired at ID16A under repeated holotomographic measurements with increasing cumulative radiation dose. The same sample was imaged multiple times with approximately 3.48 x 10^7 Gy per full holotomography scan (2000 projections, four propagation distances, 33.6 keV, 100 nm voxel size), and accumulated a dose of 1.14 x 10^8 Gy at the end of the last scan. Comparable anatomical features are preserved across repeated scans, with no visible qualitative signs of radiation-induced structural degradation observed within the explored dose range.

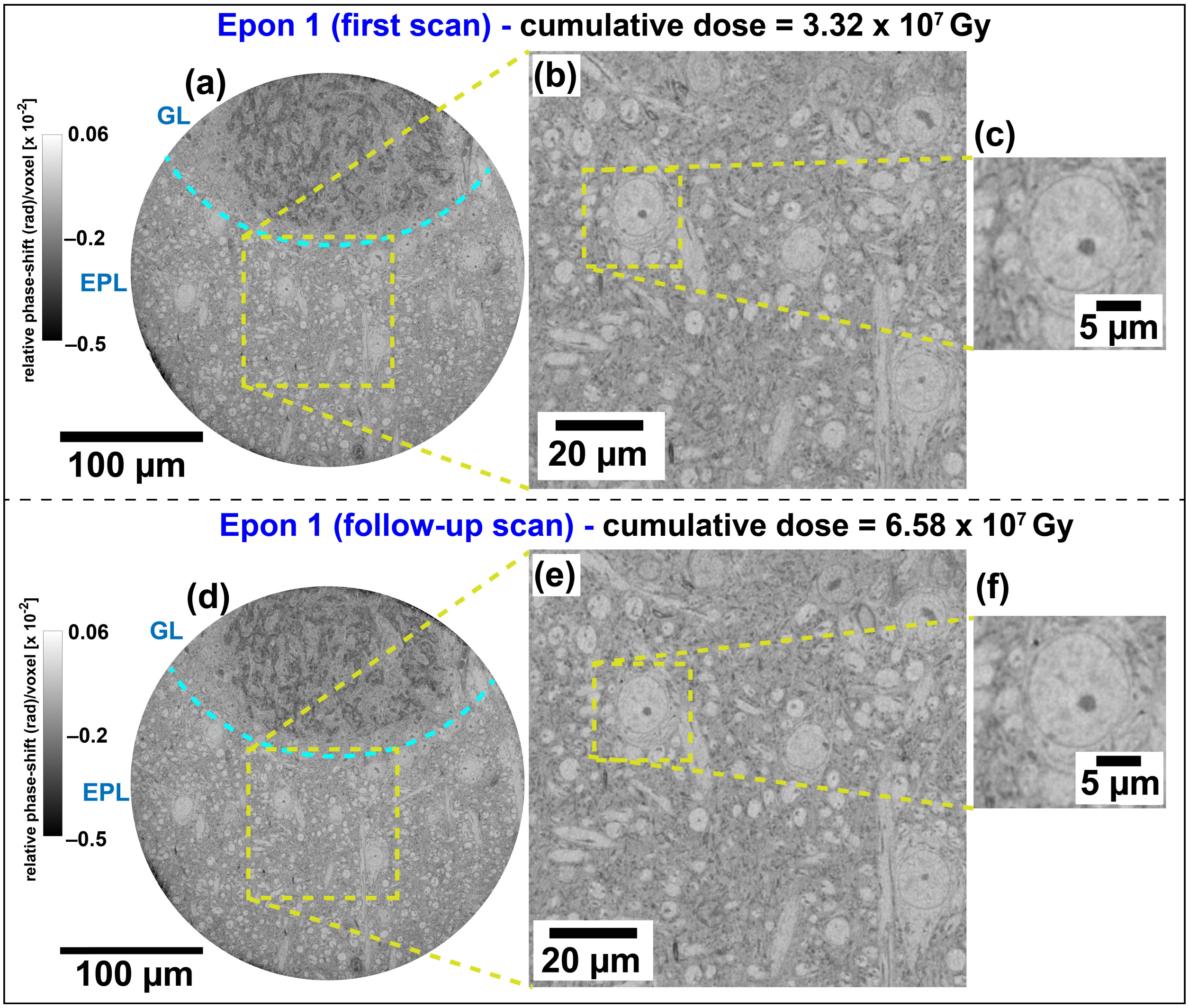

Figure S14. Radiation dose tolerance under repeated holotomographic imaging of resin-embedded brain tissue. Representative reconstructed phase-contrast slices from a resin-embedded brain tissue sample (SK185_p2) acquired at ID16A under repeated holotomographic measurements with increasing cumulative radiation dose. The same sample was imaged multiple times with approximately 3.32 x 10^7 Gy per holotomography scan (2000 projections, four propagation distances, 33.6 keV, 100 nm voxel size), and accumulated a dose of 6.58 x 10^7 Gy at the end of the last scan. No qualitative changes in tissue morphology, contrast, or emergence of radiation damage artefacts were observed across repeated acquisitions within this dose range.

	sampleID	Paper Name	Staining	Embedding	Pillar diameter (μm)	Scan-Parameters	Figures
1	SK101_p1	Epon1	rOTO	Epon	500	E=17 keV; pixel size=325 nm; exposure time= 10 ms	Fig.3
2	SK101_p3	Epon2	rOTO	Epon	500	E=17 keV; pixel size=325 nm; exposure time= 10 ms	Supp. Fig. S4
3	SK106_p1	Epon3	rOTO	Epon	500	E=17 keV; pixel size=325 nm; exposure time= 10 ms	Supp. Fig. S4
4	SK277_p1	CPD1	rOTO	CPD	500	E=17 keV; pixel size=325 nm; exposure time= 10 ms	Fig.3
5	SK271_p1	CPD2	rOTO	CPD	500	E=17 keV; pixel size=325 nm; exposure time= 10 ms	Supp. Fig. S5
6	SK282_p1	CPD3	rOTO	CPD	500	E=17 keV; pixel size=325 nm; exposure time= 10 ms	Supp. Fig. S5
7	SK287_p1	CPD4	rOTO	CPD	500	E=17 keV; pixel size=325 nm; exposure time= 10 ms	Supp. Fig. S5
8	SK197_p1	Paraffin	Unstained	Paraffin	500	E=17 keV; pixel size=325 nm; exposure time= 10 ms	Supp. Fig. S7
9	SK207_p1	CPD	Unstained	CPD	500	E=17 keV; pixel size=325 nm; exposure time= 10 ms	Supp. Fig. S7

Table S1. Summary of samples imaged using X-ray phase contrast tomography (XPCT) at beamline P14 (PETRA III, DESY). Samples were stained with heavy metals (rOTO protocol) and prepared using either critical point drying (CPD) or resin embedding (Epon). Imaging parameters include X-ray energy, effective pixel size, and exposure time.

	sampleID	Paper Name	Staining	Embedding	Pillar diameter (μm)	Scan-Parameters	Figures
1	SK185_p1	CPD1	rOTO	CPD	300	E=33.6 keV; pixel size=100 nm; exposure time= 300 ms	Fig.4
2	SK120	CPD2	rOTO	CPD	300	E=33.6 keV; pixel size=100 nm; exposure time= 300 ms	Fig.4 (averaged line-profile)
3	YY019_D1	CPD3	rOTO	CPD	150	E=33.6 keV; pixel size=60 nm; exposure time= 150 ms	Supp. Fig. S9
4	SK186_p2	Epon1	rOTO	Epon	300	E=33.6 keV; pixel size=100 nm; exposure time= 300 ms	Fig.4
5	C417F	Epon2	rOTO	Epon	300	E=33.6 keV; pixel size=60 nm; exposure time= 330 ms	Supp. Fig. S10

Table S2. Summary of samples imaged by X-ray nano-holotomography (XNH) at beamline ID16A (ESRF). Each sample was stained with heavy metals (rOTO protocol) and prepared using either critical point drying (CPD) or resin embedding (Epon). Imaging parameters include X-ray energy, effective pixel size, and exposure time.
